# Wildlife gut microbiomes of sympatric generalist species respond differently to anthropogenic landscape disturbances

**DOI:** 10.1186/s42523-023-00237-9

**Published:** 2023-04-06

**Authors:** Alexander Christoph Heni, Gloria Fackelmann, Georg Eibner, Swetlana Kreinert, Julian Schmid, Nina Isabell Schwensow, Jonas Wiegand, Kerstin Wilhelm, Simone Sommer

**Affiliations:** 1 Institute of Evolutionary Ecology and Conservation Genomics, Ulm University, 89081 Ulm, Germany; 2 Smithsonian Tropical Research Institute, Balboa, Ancón, Republic of Panama; 3 Institute of Virology, Campus Charité Mitte, Charité - Universitätsmedizin Berlin, corporate member of Freie Universität Berlin, Humboldt-Universität zu Berlin, and Berlin Institute of Health, 10117 Berlin, Germany

**Keywords:** Gut microbiome, Phylogenetics, Anthropogenic disturbance, Landscape ecology, *Proechimys semispinosus*, *Didelphis marsupialis*, *Philander opossum*, Panama

## Abstract

**Background:**

Human encroachment into nature and the accompanying environmental changes are a big concern for wildlife biodiversity and health. While changes on the macroecological scale, i.e. species community and abundance pattern, are well documented, impacts on the microecological scale, such as the host’s microbial community, remain understudied. Particularly, it is unclear if impacts of anthropogenic landscape modification on wildlife gut microbiomes are species-specific. Of special interest are sympatric, generalist species, assumed to be more resilient to environmental changes and which often are well-known pathogen reservoirs and drivers of spill-over events. Here, we analyzed the gut microbiome of three such sympatric, generalist species, one rodent (*Proechimys semispinosus*) and two marsupials (*Didelphis marsupialis* and *Philander opossum*), captured in 28 study sites in four different landscapes in Panama characterized by different degrees of anthropogenic disturbance.

**Results:**

Our results show species-specific gut microbial responses to the same landscape disturbances. The gut microbiome of *P. semispinosus* was less diverse and more heterogeneous in landscapes with close contact with humans, where it contained bacterial taxa associated with humans, their domesticated animals, and potential pathogens. The gut microbiome of *D. marsupialis* showed similar patterns, but only in the most disturbed landscape. *P. opossum*, in contrast, showed little gut microbial changes, however, this species’ absence in the most fragmented landscapes indicates its sensitivity to long-term isolation.

**Conclusion:**

These results demonstrate that wildlife gut microbiomes even in generalist species with a large ecological plasticity are impacted by human encroachment into nature, but differ in resilience which can have critical implications on conservation efforts and One Health strategies.

**Supplementary Information:**

The online version contains supplementary material available at 10.1186/s42523-023-00237-9.

## Introduction

In the Anthropocene, a plethora of challenges have forced wildlife to cope with environmental changes, such as habitat fragmentation and modification [1]. While species that are highly susceptible to changes in their environment are likely to disappear, species that are more resilient to environmental change increase in numbers and will probably become more dominant in local species assemblages [2]. The breaking apart of natural habitat into patches can have negative effects on the abundance of species and their health [3, 4]. These so-called generalist species often thrive in or at edges of altered landscapes and have contact with the matrix surrounding the habitat fragments, often leading to closer contact to humans, livestock and domesticated animals [5]. As a consequence of inhabiting a wide range of habitats, generalist species are often important pathogen reservoirs and act as vectors of zoonotic diseases, despite appearing phenotypically healthy [4]. Increasing contact rates with humans and domesticated animals, as well as the exploitation of wildlife for food or medicine, amplify the risk of spillover events in both directions [6]. However, assessing wildlife health of generalist species coping with anthropogenic disturbances can be challenging because of the problems posed in macroecological studies in assessing the internal (health) states in wild animals. Even common fitness indicators, such as body mass can point into wrong directions if land use change requires dietary shifts of the surviving, apparently adaptable resilient species [7].

The gut microbiome, the assemblage of microbes and their genomes inhabiting the gut of a host, is an integral part of an animal’s well-being and is considered as a good indicator of host health [8, 9]. Gut microbial communities carry a wide range of important functions ranging from food processing, access to nutrients, production of antibiotics, to the influence on behavior and the immune system [10], and thus can play a key role in host adaptation to rapidly changing environments [11]. Shifts in bacterial diversity beyond the ‘normal’ range can result in dysbiosis, i.e., the depletion of commensal microbes and increase in pathogenic ones [12]. Both can have negative consequences on host immunity and health. Recent studies in humans and wildlife highlighted that gut homeostasis as subject to disturbances by viral infections, facilitating co-infections and the spread of zoonotic diseases [13–15]. Dysbiosis has been linked to environmental change, with further influencing factors being stress, shifts in diet, and species assemblage on the macroecological scale [13, 14, 16–18].

Indeed, recent studies have emphasized the negative effects of landscape modifications and habitat fragmentation on host gut microbiome homeostasis. For example, the gut microbiome of howler monkeys living in fragmented areas or in captivity had a lowered bacterial diversity [19], with several follow-up studies confirming these findings in different settings and for different species [e.g. 20, 21]. However, these studies were not able to differentiate between true landscape effects and the effects of change in diet and other anthropogenic disturbances. Specifically, whether habitat fragmentation per se or the combination of habitat fragmentation with additional anthropogenic disturbances such as contact with humans, domesticated animals, and their pathogens dominate these changes and disrupts natural gut microbiota homeostasis was so far studied only in the Tome’s spiny rat (*Proechimys semispinosus*) [17]. In this study, spiny rats inhabiting isolated but otherwise undisturbed rainforest fragments showed a similar gut microbial composition as conspecifics living in undisturbed continuous forests, indicating that habitat fragmentation per se is not the driving factor behind changes in the gut microbiome. However, in general, gut microbiomes were less diverse and at the same time, individual microbiomes were more heterogeneous in spiny rats inhabiting fragmented *and* disturbed landscapes, with differences driven by microbes associated with humans and domesticated animals. However, whether these results are restricted to this species or a common feature of generalist species living in sympatry and independent of phylogeny is largely unknown, but of high importance [22].

In the present study, we analyzed the gut microbiomes of three sympatric, generalist, neotropical, mammalian species (two marsupials *Didelphis marsupialis* and *Philander opossum*, which might be renamed *Philander melanurus* in the future [23], and one rodent *P. semispinosus*) inhabiting four landscapes in central Panama with differing degrees of anthropogenic disturbances: protected continuous tropical forests, protected forested islands in the Panama Canal, nearby unprotected forested fragments embedded in an agricultural matrix and teak plantations. The three study species represent abundant generalists living in rainforests with a high tolerance for habitat modifications [24, 25]. *P. semispinosus* is a strictly terrestrial rodent that mainly feeds on fruits, seeds and mycorrhiza and occupies rather small home ranges, often representing one of the most abundant mammalian species within its geographic range [26, 27]. *D. marsupialis* is a common terrestrial, omnivorous marsupial with a large home range [28, 29] and *P. opossum* is a semi-arboreal marsupial with an omnivorous diet including fruits, vertebrates, and invertebrates with a home range of 0.34 ha in Panama [29–31]. All three species represent important pathogen reservoirs and hosts for zoonotic diseases of clinical relevance, caused by *Trypanosoma* [32], *Hepacivirus* [24], mammalian delta virus [33], *Schaalia* [34] and Venezuelan equine encephalitis virus [35]. *P. semispinosus* and *D. marsupialis* are known to be consumed by humans as a source of protein, making them particularly interesting in terms of their potential roles in spillover events [36, 37]. This highlights the importance of understanding any potential health impacts anthropogenic disturbances may have on these species. With this study design, we aimed to investigate (1) to what degree, if any, do host species identity (i.e., phylogeny) and landscape modifications shape the gut microbiomes of three sympatric, generalist species; (2) to which extent do the host species differ in their resilience, i.e. responses to different degrees of anthropogenic disturbances; and finally (3) which gut bacteria are involved in driving any changes, and are they similar for all three host species? Gaining a better understanding of the gut microbial response to anthropogenic disturbances is of high importance in maintaining wildlife health, especially in generalist species that can act as vectors transmitting diseases among wildlife, but also livestock, domestic animals and ultimately humans.

## Results

### Species community and abundance pattern

During three field seasons, 1523 animals of 16 different species were captured in 28 study sites distributed across four landscapes (protected continuous tropical forests (=C), protected forested islands in the Panama Canal (=I), nearby unprotected forested fragments embedded in an agricultural matrix (=A) and teak plantations (=P)) differing in degree of anthropogenic disturbance in Panama (Additional file 1: Figs. S1 and S2). The three most common animals captured were *P. semispinosus* (*n* = 1235), *D. marsupialis* (*n* = 137) and *P. opossum* (*n* = 72). *P. semispinosus* and *D. marsupialis* were captured in all four landscapes, while *P. opossum* was absent on landscape I. Fecal samples from 397 *P. semispinosus*, 104 *D. marsupialis*, and 68 *P. opossum* were available for microbiome investigation (Additional file 2: Table S1).

### Gut bacterial composition of the three generalist species

**Fig. 1:**
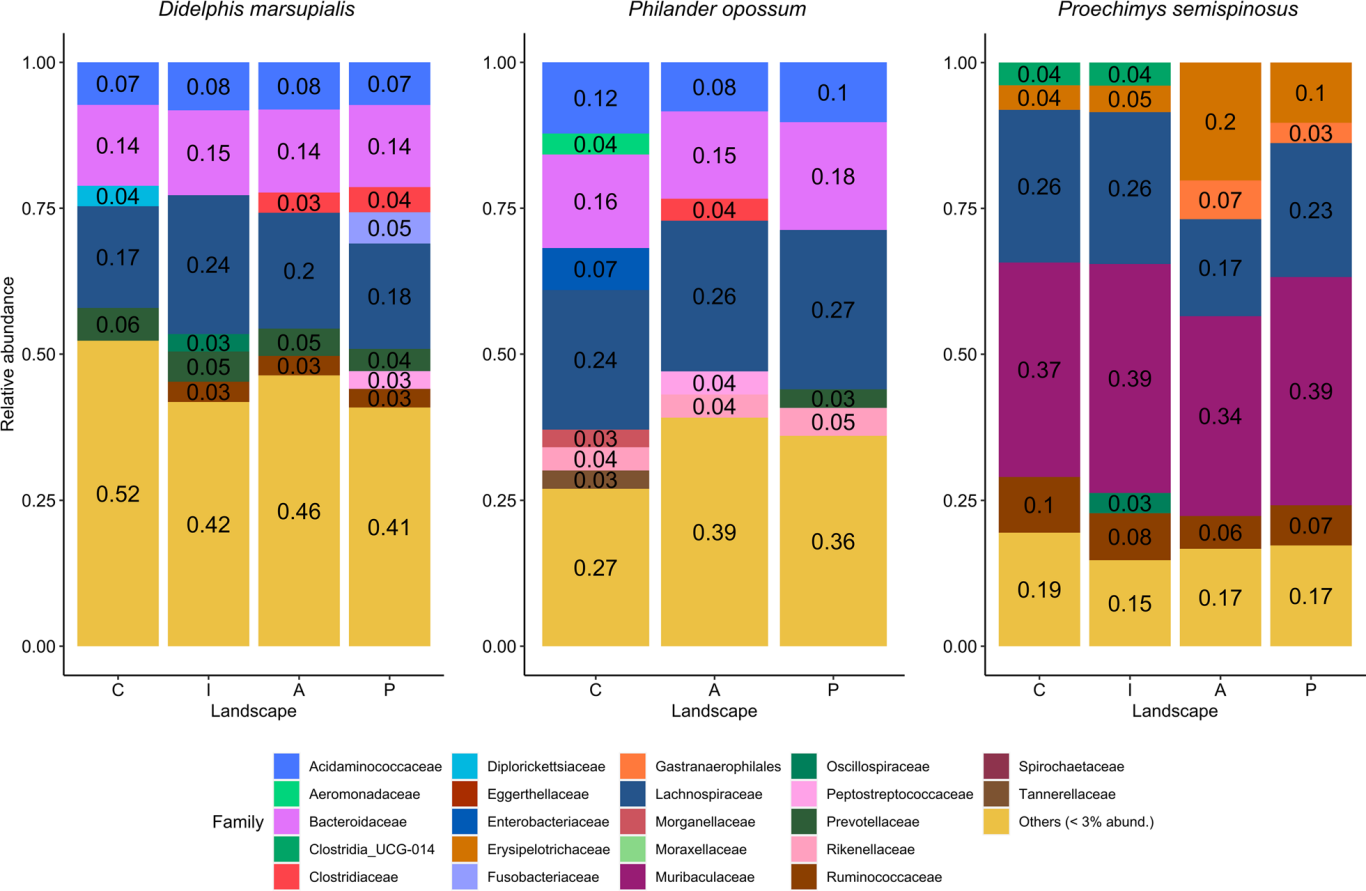
Gut bacterial composition (at the family level) of the two marsupials (*D. marsupialis*, *P. opossum*) and the spiny rat (*P. semispinosus*). Details on the landscapes C, I, A and P are provided in the methods and their locations are shown in Additional file 1: Fig. S1. Note that *P. opossum* was not captured in landscape I

After sequencing the 16S rRNA gene from fecal matter and rarefication a total of 6404 different bacterial ASVs were identified in the 569 samples kept after quality filtering (Additional files 1 and 2: Fig. S3 and Table S1). The two marsupial species, *P. opossum* and *D. marsupials*, had 1454 ASVs in common, whilst the ground-dwelling species *D. marsupialis* and *P. semispinosus* shared 445 ASVs, and the microbiome of *P. opossum* and *P. semispinosus* overlapped in only 252 ASVs (Additional file 1: Fig. S4). The two opossum species shared more dominant bacterial families with each other than with the rodent species, *P. semispinosus* (Fig. 1). In the marsupials, the gut microbiomes were dominated by the bacterial families *Lachnospiraceae*, *Bacteroidaceae* and *Acidaminococcacae*. Considering less frequent bacterial families in *D. marsupialis*, *Diplorickettsiaceae* were mainly found in landscape C, while *Ruminococcaceae* were mainly present in the landscapes I, A and P. *Oscillospiraceae* were detected in landscape I, while *Clostridiaceae* were mainly present in the disturbed landscapes A and P. *Fusobacteriaceae* and *Peptostreptococcaceae* were mainly detected in landscape P. In *P. opossum*, the gut microbiomes of individuals trapped in landscape C were characterized by the presence of *Clostridia_UCG-014*, *Enterobacteriaceae* and *Morganellacea,* while *Peptostreptococcaceae* and *Clostridiaceae* were mainly found in the gut microbiome of individuals inhabiting landscape A and the presence of *Prevotellaceae* was characteristic for landscape P. *Muribaculaceae* (formerly known as *S24-7* [38]), *Lachnospiraceae* and *Erysipelotrichaceae* were the dominant bacterial families in the gut of *P. semispinosus*. The gut microbial composition of *P. semispinosus* differed between the landscapes C and I by the addition of *Oscillospiraceae* in landscape I. Rodents trapped in the landscapes A and P both lacked *Clostridia_UCG-014* but harbored *Gastranaerophilales* instead (Fig. 1).

### Gut bacterial composition is shaped by both host phylogeny and landscape type

**Fig. 2:**
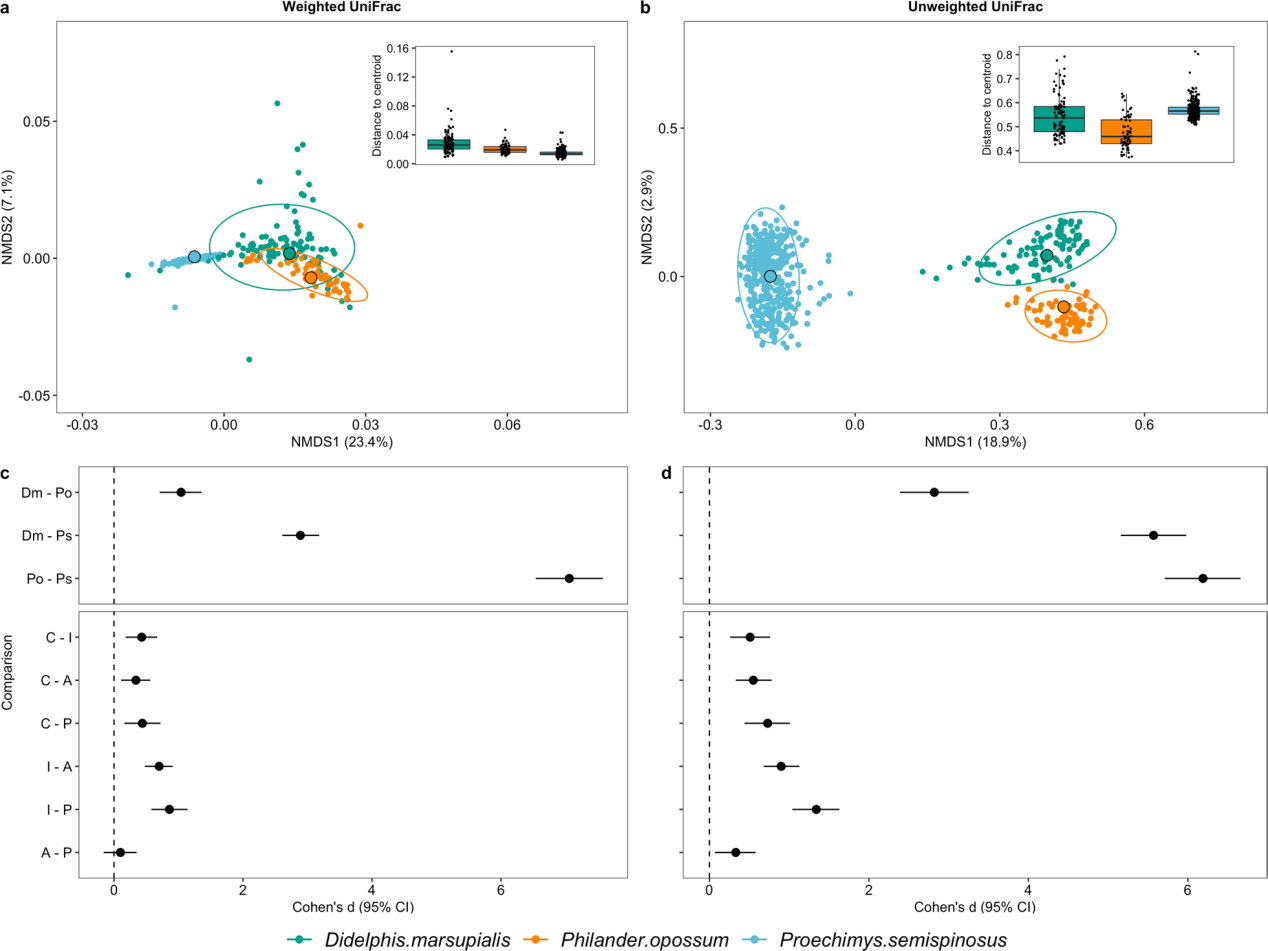
Differences in beta diversity between three sympatric generalist species, the marsupials *D. marsupialis* (Dm, green), *P. opossum* (Po, orange), and the rodent *P. semispinosus* (Ps, blue). The top row shows NMDS plots of **a** weighted and **b** unweighted UniFrac distances. The bottom row **c**, **d** displays corresponding forest plots with Cohen’s *d* effect sizes highlighting the phylogenetic signal and landscape effects, i.e. the different levels of anthropogenic disturbance. Details on the landscapes C, I, A and P are provided in the methods and their locations are shown in Additional file 1: Fig. S1

We first investigated if species identity and/or landscape type shaped gut microbial beta diversity (both composition and dispersion) across all three sympatric species. Using one model with host species identity and landscape type as fixed factors per distance matrix (weighted and unweighted UniFrac distances), both species identity (PERMANOVA: weighted Unifrac: df = 2, R^2^ = 0.23, *p* ≤ 0.001; unweighted UniFrac: df = 2, R^2^ = 0.21, *p* ≤ 0.001) and landscape type had significant effects on gut microbial composition (PERMANOVA: weighted Unifrac: df = 3, R^2^ = 0.02, *p* ≤ 0.001; unweighted UniFrac: df = 3, R^2^ = 0.02, *p* ≤ 0.001; Fig. 2a, b). Based on effect sizes (Cohen’s *d*), the effect of phylogeny was larger than the effect of landscape type (range based on weighted UniFrac distances: host phylogeny: 1.04–7.06, landscape: 0–0.76; range based on unweighted UniFrac distances: host phylogeny: 2.82–6.19, landscape: 0.33–1.34; Fig. 2c, d). Moreover, pairwise comparisons of each host species pair showed the smallest differences in gut microbial composition between both marsupials, and larger differences between either marsupial and the rodent (Fig. 2c, d). The effect size for pairwise comparisons of landscape type showed that each landscape was distinct from one another, except for the comparison between the disturbed landscapes A and P using weighted UniFrac distances (Fig. 2c). In addition to the bacterial composition, gut microbial dispersion was also significantly affected by both host species identity (weighted UniFrac: df = 2, F value = 174.93, *p* ≤ 0.001; unweighted UniFrac: df = 2, F value = 122.47, *p* ≤ 0.001, Fig. 2a, b) and landscape type (weighted UniFrac: df = 3, F value = 29.62, *p* ≤ 0.001; unweighted UniFrac: df = 2, F value = 33.38, *p* ≤ 0.001).

### Species-specific effects of landscape type on gut microbial alpha and beta diversity of sympatric generalist species

**Fig. 3:**
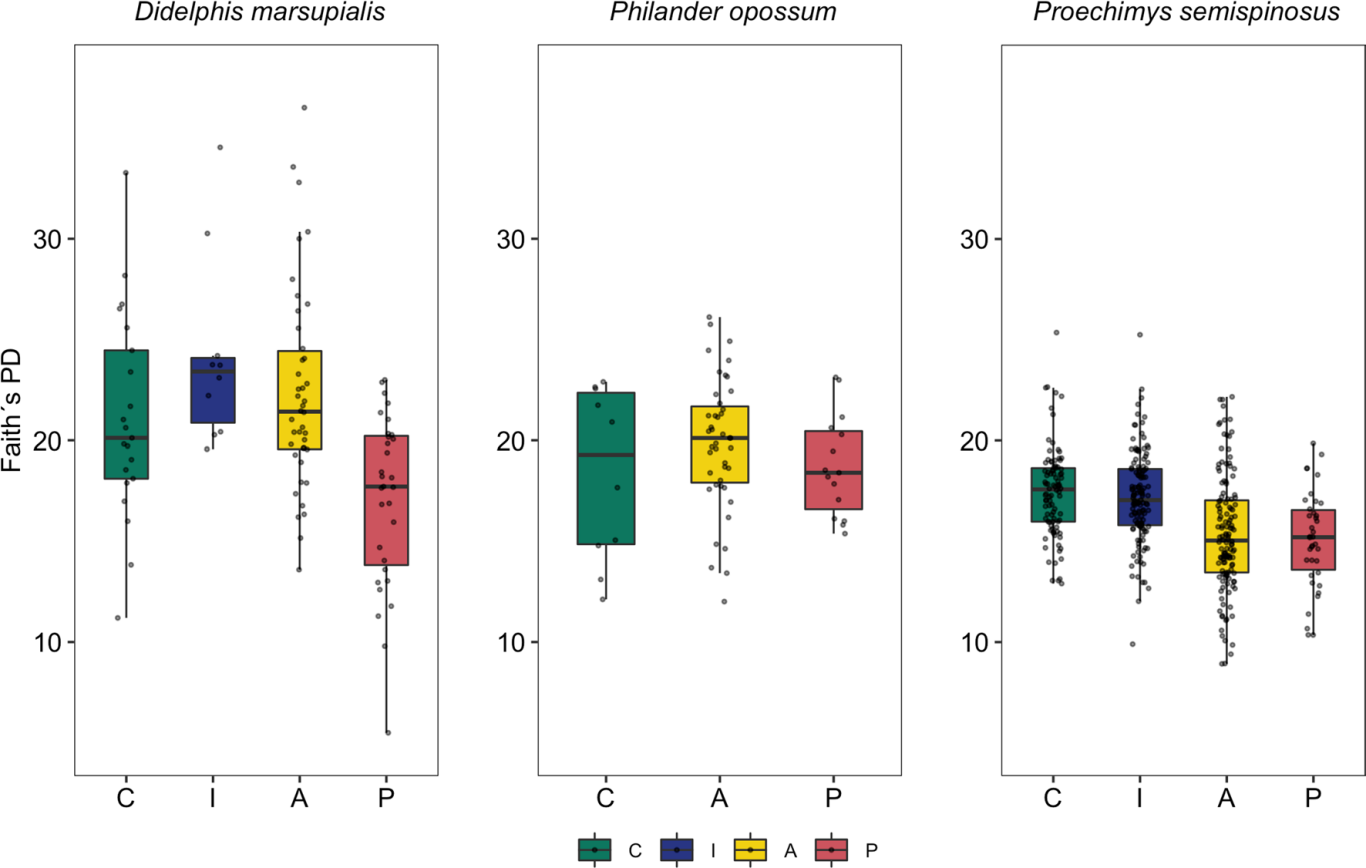
Landscape effects on alpha diversity (measured as Faith´s PD) of three sympatric generalist species, the marsupials *D. marsupialis* and *P. opossum*, and the spiny rat *P. semispinosus*. Individuals were trapped in protected continuous tropical forests (landscape C, green); protected forested islands in the Panama Canal (landscape I, blue); in the nearby unprotected forested fragments embedded in an agricultural matrix (landscape A, yellow) and in teak plantations (landscape P, red)

**Fig. 4:**
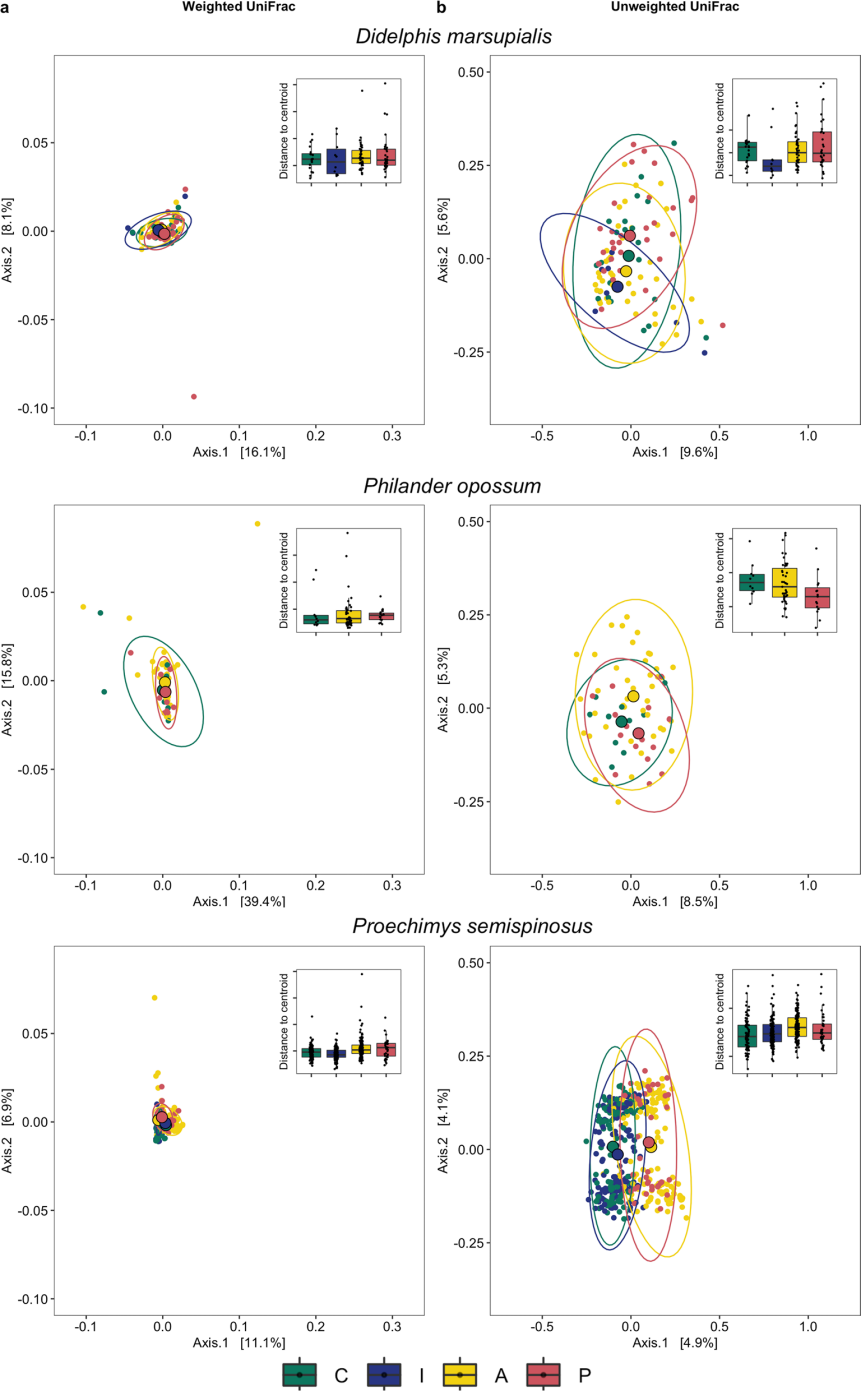
NMDS plots visualizing landscape effects on beta diversity. Beta diversity is measured by **a** weighted and **b** unweighted UniFrac distances in the three sympatric generalist species, the marsupials *D. marsupialis* (top) and *P. opossum* (middle), and the spiny rat *P. semispinosus* (bottom). Distances to the group centroids are depicted in the inserted graphs in the top right corners and ellipses indicate 95% confident intervals. Individuals were trapped in protected continuous tropical forests (landscape C, green); protected forested islands in the Panama Canal (landscape I, blue); in the nearby unprotected forested fragments embedded in an agricultural matrix (landscape A, yellow) and in teak plantations (landscape P, red)

To determine if landscape type had similar effects on gut microbial alpha and beta diversity across three different, yet sympatric species, we analyzed the effect of landscape type on gut microbial alpha diversity, composition, and dispersion for each species separately. In the ground-dwelling marsupial *D. marsupialis*, all alpha diversity metrics were significantly impacted by landscape type (Additional file 2: Table S2), with individuals inhabiting landscape P having a significantly lower gut microbial alpha diversity than those living in the other three landscape types (Additional file 2: Table S2, depicted for Faith’s PD, Fig. 3). Beta diversity based on weighted UniFrac distances did not show a significant effect of landscape type on gut microbial composition and dispersion (PERMANOVA: df = 3, R^2^ = 0.04, *p* = 0.1; PERMDISP: df = 3, F value = 0.43, *p* = 0.74, Fig. 4). However, using unweighted UniFrac distances, gut microbial composition (but not dispersion; PERMDISP: df = 3, F value = 1.22, *p* = 0.30) was significantly affected by landscape type (PERMANOVA: df = 3, R^2^ = 0.04, *p* ≤ 0.01, Fig. 4) with plantations (landscape P) clearly differing from the other three landscapes C, I and A based on pairwise comparisons (Additional file 2: Table S2).

In the second, partially arboreal, marsupial species *P. opossum* that was never captured on landscape I, landscape type did not have a significant effect on Faith’s PD (Additional file 2: Table S3, Fig. 3) or on gut bacterial community dispersion (PERMDISP: weighted UniFrac: df = 2, F value = 0.16, *p* = 0.21; unweighted UniFrac: df = 2, F value = 2.72, *p* = 0.07). It did, however, significantly affect gut microbial composition with regards to unweighted UniFrac distances (PERMANOVA: df = 2, R^2^ = 0.04, *p* ≤ 0.01, Fig. 4), with all landscapes differing from one another based on post-hoc pairwise comparisons, but not with regards to weighted UniFrac distances (PERMANOVA: df = 2, R^2^ = 0.04, *p* = 0.21; Fig. 4, Additional file 2: Table S3).

In the ground-dwelling, rodent species *P. semispinosus*, landscape type had a significant effect on Faith’s PD (Additional file 2: Table S4, Fig. 3) and pairwise comparisons showed that each landscape type significantly differed from one another, except for the two protected landscapes C and I as well as the two unprotected landscapes A and P (Additional file 2: Table S4, Fig. 3). Similarly, gut microbial beta diversity (composition and dispersion) was significantly impacted by landscape type, regardless of distance metric (weighted UniFrac: PERMANOVA: df = 3, R^2^ = 0.07, *p* ≤ 0.001; PERMDISP: df = 3, F value = 13.21, *p* ≤ 0.001, pairwise post-hoc test Additional file 2: Table S4; unweighted UniFrac: PERMANOVA: df = 3, R^2^ = 0.05, *p* ≤ 0.001; PERMDISP: df = 3, F value = 7.17, *p* ≤ 0.001, Additional file 2: Table S4), with a clear separation between protected (landscapes C and I, without contact to humans, livestock and domestic animals) and unprotected (landscapes A and P, with contact to humans, livestock and domestic animals) landscape types (Fig. 4). Thus, the gut microbiomes of generalists are affected by anthropogenic disturbances, but sympatric species show species-specific differences in their responses.

### Landscape-driven species-specific taxonomic differences in gut microbial beta diversity

To gain a better understanding of which bacterial taxa are driving the differences in beta diversity between landscape types within each host species, we ran three different differential abundance analyses, one for each species, using ANCOM [39]. Because this is a pairwise test, we grouped together individuals from landscape types that did not significantly differ in the aforementioned beta diversity analyses, i.e., showed similar responses to landscape effects on beta diversity.

**Fig. 5:**
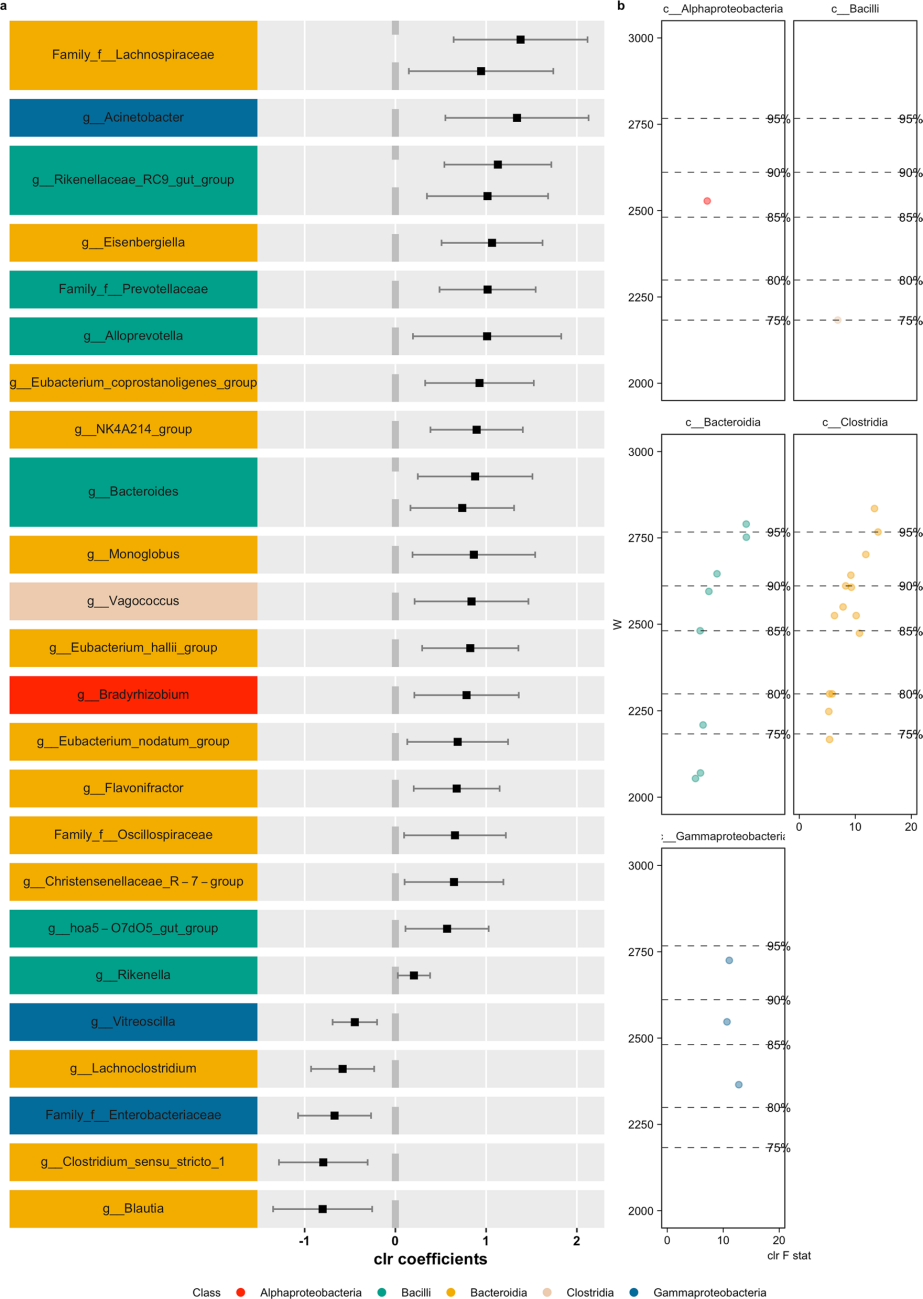
Differentially abundant ASVs (using ANCOM) in pairwise comparisons with landscape groupings based on similar responses in beta diversity to landscape modifications (Fig. 4) in *D. marsupialis* comparing landscape C + I + A against P. Colors represent the different bacterial classes. **a** Differential abundant ASVs according to the taxonomical assignment; **b** Volcano plot of differential abundant ASVs, depicting the F statistics and W-value

**Fig. 6:**
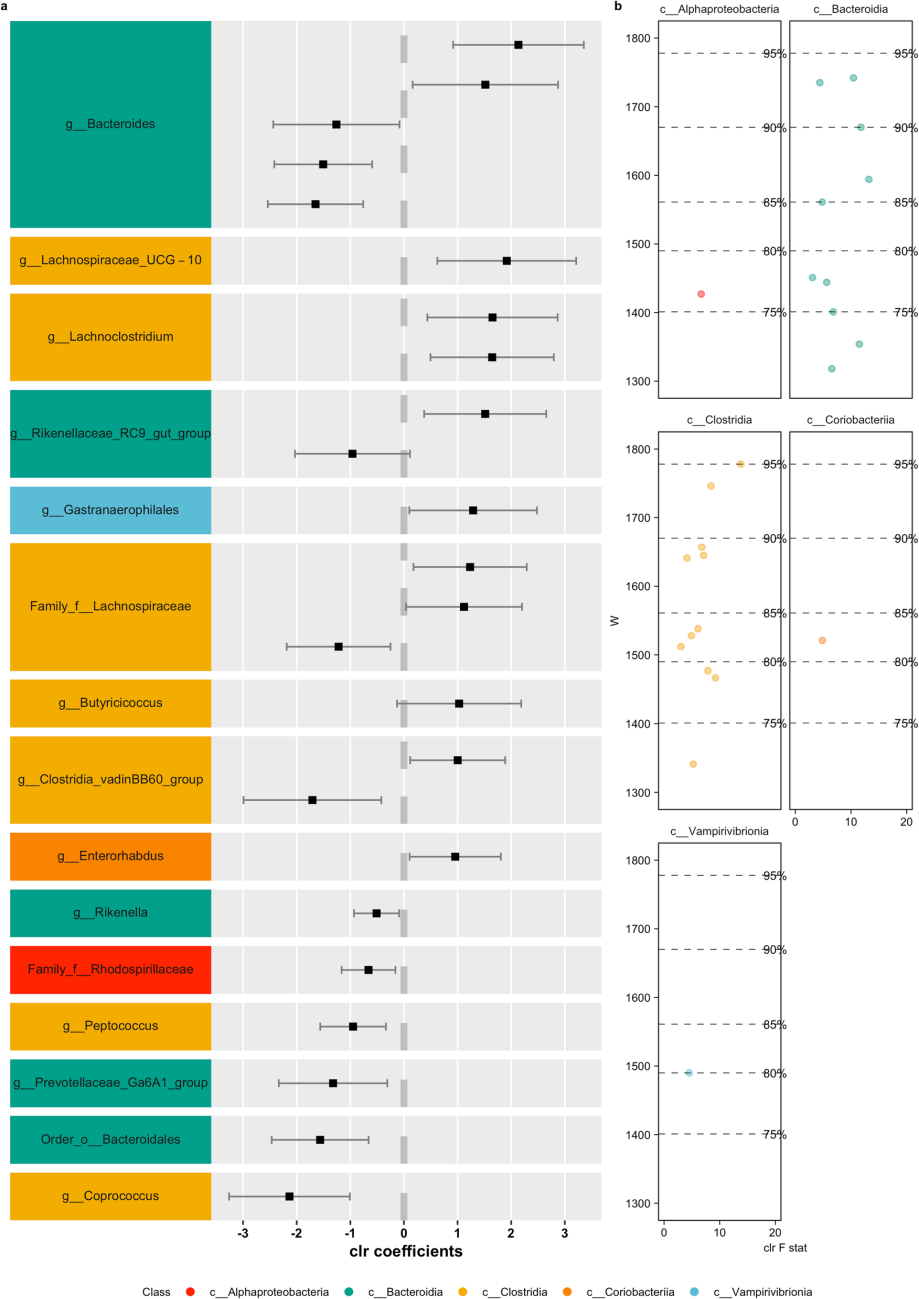
Differentially abundant ASVs (using ANCOM) in pairwise comparisons with landscape groupings based on similar responses in beta diversity to landscape modifications (Fig. 4) in *P. opossum* comparing landscape C against A + P. Colors represent the different bacterial classes. **a** Differential abundant ASVs according to the taxonomical assignment; **b** Volcano plot of differential abundant ASVs, depicting the F statistics and W-value

**Fig. 7:**
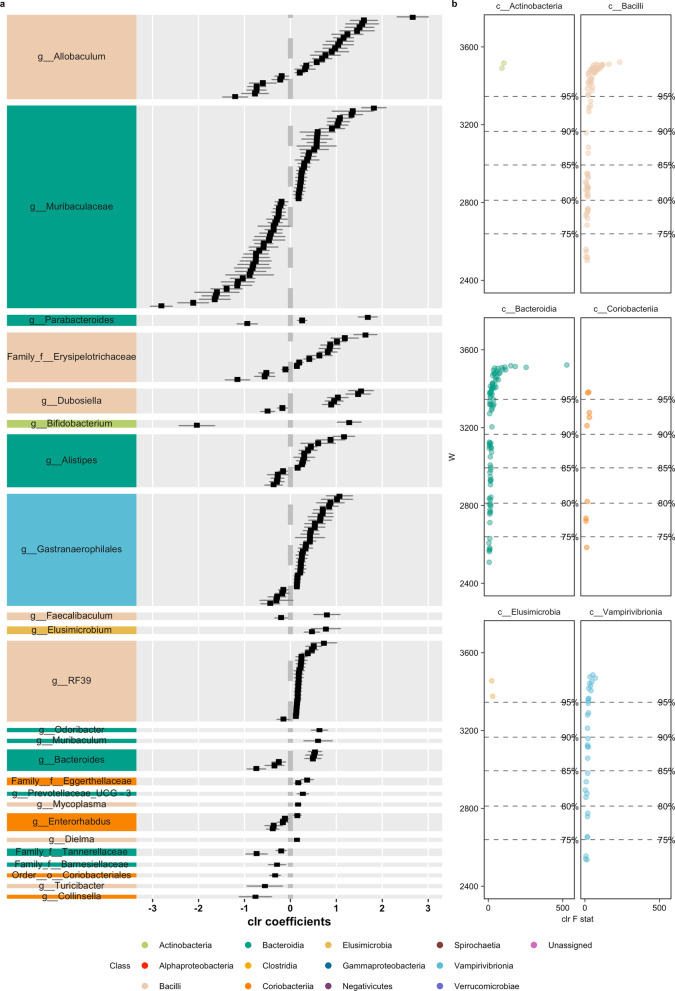
First half of differentially abundant ASVs (using ANCOM) in pairwise comparisons with landscape groupings based on similar responses in beta diversity to landscape modifications (Fig. 4) in *P. semispinosus* comparing landscape C + I against A + P. Results split into two graphs. Colors represent the different bacterial classes. **a** Differential abundant ASVs according to the taxonomical assignment; **b** Volcano plot of differential abundant ASVs, depicting the F statistics and W-value

**Fig. 8:**
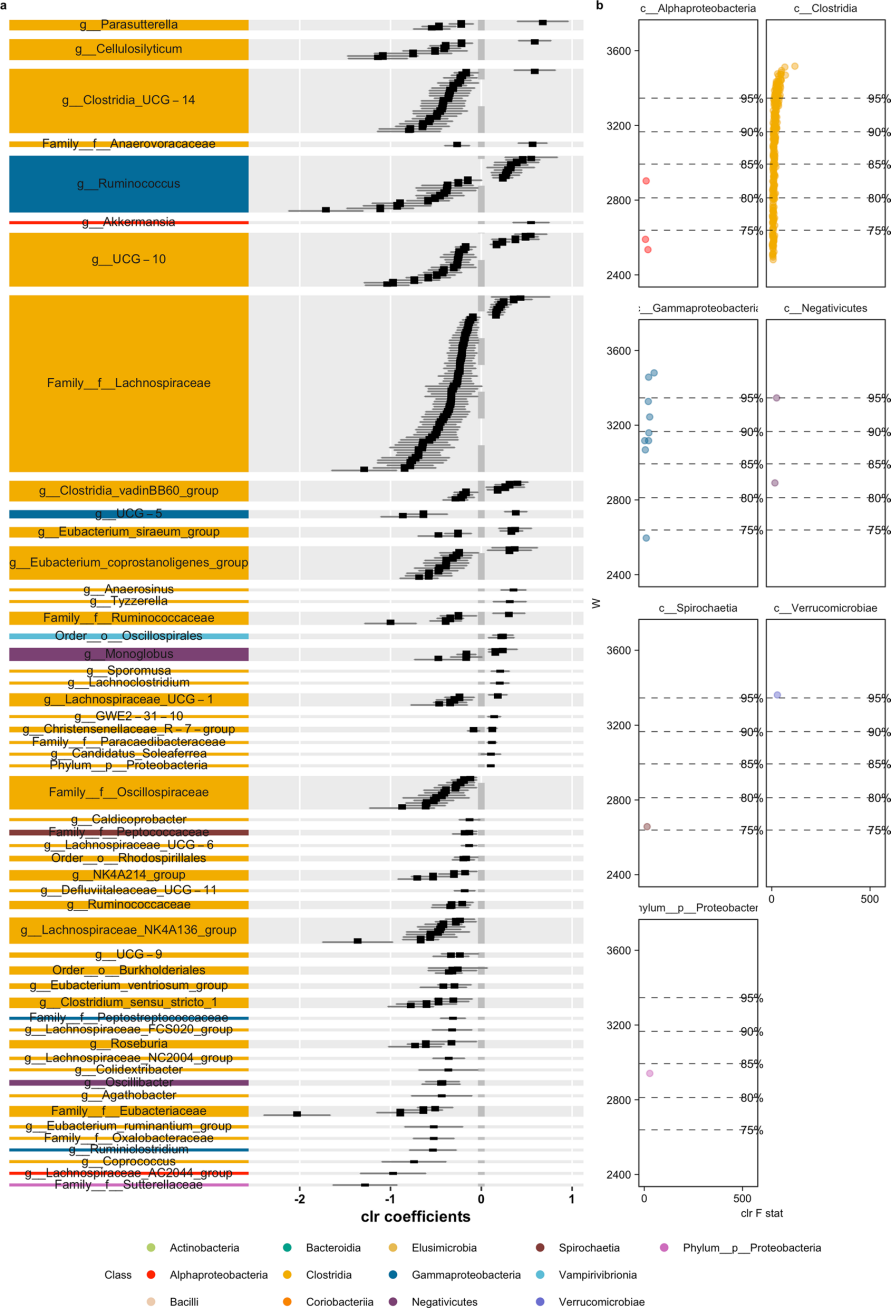
Second half of differentially abundant ASVs (using ANCOM) in pairwise comparisons with landscape groupings based on similar responses in beta diversity to landscape modifications (Fig. 4) in *P. semispinosus* comparing landscape C + I against A + P. Results split into two graphs. Colors represent the different bacterial classes. **a** Differential abundant ASVs according to the taxonomical assignment; **b** Volcano plot of differential abundant ASVs, depicting the F statistics and W-value

First, for *D. marsupialis*, we compared gut microbiomes from individuals inhabiting landscapes C + I + A against P and identified 27 differentially abundant ASVs from the bacterial classes *Alphaproteobacteria*, *Bacilli*, *Bacteroida*, *Clostridia,* and *Gammaproteobacteria* (Fig. 5). Second, for *P. opossum*, we compared the gut microbial communities of individuals living in the control landscape C against those from the disturbed landscapes A and P (this species was not captured on landscape I). Because no clear separation of the microbial alpha- or beta-diversity based on landscape type was observed, we decided to use our hypothesis-driven assumption, comparing the two disturbed landscapes against the protected control (landscape C). In doing so, we detected 24 differentially abundant ASVs from the bacterial classes *Alphaproteobacteria*, *Bacteroida*, *Clostridia*, *Coriobacteriia* and *Vampirivibrionia* (Fig. 6). Lastly, for *P. semispinosus*, we compared the gut microbial compositions of rodents inhabiting the protected landscapes C and I to those living in the unprotected landscapes A and P and found 481 differentially abundant ASVs from the bacterial classes *Actinobacteria*, *Alphaproteobacteria*, *Bacilli*, *Bacteroida*, *Clostridia*, *Coriobacteriia*, *Elusimicrobia*, *Gammaproteobacteria*, *Negativicutes*, *Spirochaetia*, *Vampirivibrionia*, and *Verrucomicrobiae* (Figs. 7 and 8).

Among the ASVs identified were representatives, such as Chrisensenellaceae R-7 group (*D. marsupialis*), Chlostridium senu stricto I (*D. marsupialis*), Rikenella (*D. marsupialis*), Vagococcus (*D. marsupialis*), Butyricoccus (*P. opossum*), Coprococcus (*P. opossum*), Peptococcus (*P. opossum*), Rikenella (*P. opossum*), Bacteroides (*P. semispinosus*), Allobaculum (*P. semispinosus*) and Dubsosiella (*P. semispinosus*).

## Discussion

### Impact of habitat change on species with high ecological plasticity

In the Anthropocene, the selection pressures faced by species worldwide have changed to favor species possessing ecological plasticity, with a higher tolerance to anthropogenically modified environments [40], the so-called generalist species [41]. Failure to adapt can especially impact animals with narrow ecological niches, leading to changes in the composition of animal communities [42]. Increases in the abundance of generalists in disturbed landscapes at the cost of the decline of less resilient species can be accompanied by negative consequences for the ecosystem and increase the risk of disease spread and spillover [43]. As anthropogenic selection pressures will affect these pathogen reservoirs and disease vectors best adapted to living in close proximity to human settlements, studies on generalists’ role in emerging infectious diseases from wildlife and their adaptability to global environmental changes are key in order to understand the threat they are under and the threat they may pose [4, 44]. In fact, deforestation is considered one of the main drivers of zoonotic diseases [45], yet, current reforestation programs often rely on a few tree species or even monocultures, such as eucalyptus or teak plantations, which might accelerate the risk [46].

### Phylogeny and landscape influence the microbiome of sympatric species

In central Panama, we investigated four landscapes differing in their degree of anthropogenic disturbance which have been shown to have severe impacts on different levels of diversity: species assemblages and abundance patterns [24, 42, 47], pathogen diversity and host infections [18, 25, 48], neutral and adaptive genetic diversity [18, 25] along with gut microbial community patterns [17, 18] differed according to landscape type. As a crucial factor in host health, the gut microbiome has been put forward as a potentially vital component in helping wildlife to adapt to fast-paced global changes [11]. However, whether this holds true for a wider range of species facing the same degrees of anthropogenic disturbance was the focus of the present study.

Here, we showed that three sympatric, generalist species faced with the same landscape-level anthropogenic disturbances each responded differently with regards to gut microbial diversity and that the gut microbiome of sympatric species is determined by both phylogeny and landscape. We observed species-specific gut microbial responses to different landscape types, with *P. semispinosus* showing the highest sensitivity to proximity to humans. In *D. marsupials*, however, only the landscape with a combination of human contact, fragmentation, and complete changes in forest type (from lowland tropical rainforest to teak plantation) led to changes in the gut microbiome. In contrast, *P. opossum*’s gut microbiome showed the highest resilience, without a distinct pattern of change in the gut microbial alpha diversity or beta dispersion. These species-specific responses were mirrored in our differential abundance analyses that showed the highest number of differentially abundant ASVs in *P. semispinosus* versus the two other species. The gut microbiomes of our three analyzed species were affected by both species identity and landscape type, which is supported by other studies [49, 50].

Species identity can influence the gut microbiome not only because of species-specific genetic constitutions, but also because species identity determines suitable environment, food preferences and processing potential, pathogen resistance, as well as adaptive potential [51]. Similarly, landscape types can differ in food availability as well as exposure to pathogens, pesticides, and different plant and animal communities. The gut microbiomes of the two opossum species were more similar to one another than they were to the spiny rat. A similar trend was shown in large African mammals, where gut microbial composition was closely correlated with phylogeny [52], as did the gut microbiome in some primates [53]. While some studies show the effect of species identity and environment on several species’ gut microbiomes, [22, 49], they are often limited to extreme environmental situations [22, 54], thus only comparing extremely contrasting conditions for the animals. However, landscape plays a large role in shaping the microbial community in humans and apes [55], and habitat fragmentation can have an influence on the gut microbiome through dietary changes [56]. Similar results have been discovered in different howler monkey species, where host species was a key predictor of the gut microbiota, but forest type, habitat, and season explained species-specific variances [57].

### Only extreme habitat perturbation changes *D. marsupialis* gut microbiomes

Analyzing the three species separately showed that changes in gut microbial composition in *D. marsupialis* occurred only in individuals inhabiting the most disturbed landscape, namely teak plantations. Teak plantations are defined by a different forest type in addition to being fragmented and embedded in a matrix with human contact. Because they are monocultures, teak plantations can alter the food availability of the inhabiting animals and provide a harsher environment, e.g. due to increased risk of forest fires [58]. Additionally, the conversion of natural habitats to teak plantations exposes animal communities to changes in microbial soil composition [59]. *D. marsupialis* individuals living in these plantations either need to adapt to these changes and the potential stress associated with them or use the plantations as a corridor for travel.

### High resilience of *P. opossum* gut microbiomes to anthropogenic changes

The second marsupial, *P.* opossum, however, showed a different gut microbial response. Despite only occurring in three of the four sampled landscapes, *P. opossum* individuals showed little change in gut microbial composition with no clear patterns across the different landscape types. Changes in gut microbial composition between individuals inhabiting all three landscapes were only observed when using unweighted UniFrac distances, indicating that rare ASVs are likely responsible for these changes. As a semi-arboreal habitat generalist, *P. opossum* individuals could escape bacterial taxa introduced by humans and livestock to soils. This could explain the weakened gut microbial response compared to the more closely-related *D. marsupialis*. Further risk of coming into contact with microbes from humans or domesticated animals is reduced because part of *P. opossum*'s diet consists of insects [60], meaning this host might be less exposed to bacteria in the ground or associated with plants compared to, say, herbivorous hosts. The microbiomes of animals and their environment are linked and shape each other in both directions [61, 62]. Still, the fact that prolonged isolation and fragmentation possibly lead to local extinction (as seen by their absence on islands) could indicate that *P. opossum* is not insensitive to landscape changes. Because islands provide the harshest matrix based on accessibility and usability as a corridor for movement, their extinction on islands could be due to a past bottleneck event. However, there are interesting dynamics of opossum species in the Panama Canal area with local disappearance as well as re-colonization of some small islands close to the mainland, thus opening the possibility for re-colonization [63].

### Large impact of human vicinity on *P. semispinosus* gut microbiomes

The gut microbiome of the common *P. semispinosus* is very sensitive to human contact, as was shown by the reduced microbial diversity and shifts in composition, driven by bacterial taxa associated with humans and domesticated animals, as well as increased dispersion of the bacterial community in individuals from fragments surrounded by agriculture [17]. All these features were not detected in individuals sampled in continuous forests or protected forested islands [17]. While the impact of protected landscape types with no human contact (continuous forests and islands) versus fragmented landscapes with human contact (forest fragments embedded in an agricultural matrix) on *P. semispinosus* gut microbiomes have been discussed elsewhere [17], the apparent similar behavior of the gut microbiome from individuals living in teak plantation to those inhabiting forest fragments is noteworthy. Contrarily to forested fragments, teak plantations also differ in the natural assembly of trees, with the dominant species being the from Asia introduced teak tree (*Tectona grandis*). The gut microbiome of *D. marsupialis* was only impacted in this landscape type, which we consider to be the most extreme landscape type, and therefore, we expected an additional impact on the *P. semispinosus* gut microbiome on top of the changes caused by the forest fragments. However, the lack of a compounded impact could mean two things: (1) individuals from fragmented forests already display the most extreme gut microbial perturbation tolerable by this species, or (2) teak plantations provide a suitable forest habitat for *P. semispinosus*, despite being monocultures and lacking trees typical for rainforests. It also indicates that the presence of humans and domesticated animals in the matrix alone shapes the microbiome, and not fragmentation or forest tree composition per se.

### Human-vicinity-associated ASVs drive gut microbial changes

Differential abundance analysis revealed a large difference in the number of ASVs determined to be differently abundant between the landscapes when considering each of the three species separately. While for the two opossum species only a handful of differentially abundant bacterial taxa were detected, many were discovered for *P. semispinosus*. In general, detected bacteria often showed similarity to ones characterized in more detail in human or domesticated animal microbiomes (e.g. *Allobaculum* [64]). Most of the detected ASVs have already been discussed [17], theorizing that a large portion of the identified bacteria could have been introduced by humans and their domesticated animals. In addition, in *D. marsupialis*, we detected ASVs assigned to the genus *Vagococcus*, which was first isolated from chicken feces [65], animals that *D. marsupials* are known to get into close contact with because they prey on their eggs [36]. Landscapes A and P are in close vicinity to human settlements, therefore uptake of these human-driven taxa into their gut microbiomes is not unexpected and could cause harm to the host by changing gut microbial composition.

### Potential consequences of microbiome changes induced by anthropogenic landscape disturbance

*D. marsupialis* is known to be consumed by humans, both for nutritional and cultural reasons [36]. Thus, not only proximity, but also direct contact with humans can be an important pathway for pathogen transmission, and a perturbed microbiome might have negative effects on host immunity, given the microbiome’s interplay and crosstalk with the host immune system [66]. Altered microbial communities can facilitate pathogen infection [48] and vice versa pathogen infection can change the microbiome [13–15]. These perturbed microbiomes further increase the risk of horizontal gene transfer which could lead to pathogenic bacteria [67]. Close contact between humans and domesticated animals can be dangerous for wildlife and humans, as demonstrated with the spillover of Nipah virus, which made the jump from flying foxes to humans through pigs as an intermediate host [68, 69]. Because land-use change can cause pandemics and the emergence of new diseases [70] and because rodents and marsupials represent a significant zoonotic disease risk in the future [71], these findings become even more important regarding the potential for the origins and emergence of zoonotic diseases, and future studies will reveal how these microbiome changes impact animal’s fitness in detail. Moreover, the loss of microbial diversity has been recognized as a potential threat to the discovery of new drugs or therapeutics in the field of microbial biotechnology [72].

## Conclusion

Overall, we could show that the gut microbiomes of sympatric species inhabiting landscapes with differing degrees of anthropogenic disturbance are mainly shaped by host species identity, with landscape type playing a smaller but significant role. Interestingly, there is a species-specific gut microbial response to landscape type, indicating that findings from one species cannot always be generalized to other species, even not to those living in the same habitat or to those that are closely related. This shows that these generalists' gut microbiomes are sensitive to landscape-level changes, a fact not detected by biodiversity monitoring of vertebrates, and that these changes are not uniform across host species. Thus, even for generalists, environmental changes can pose a big impact, which is an important finding as this might directly cause consequences and the risk of emerging zoonoses not only for wildlife health, but also for the health of domesticated animals intended for human consumption, and for humans themselves.

## Material and methods

### Study area and sampling

This study was carried out in the Panama Canal area, Panama. Animals were captured in 28 study sites grouped into four different landscapes based on their degree of anthropogenic disturbance (Additional file 1: Fig. S1, map created with ggmap [73]): (1) continuous rainforest (=C, five capture sites), i.e., undisturbed and protected lowland tropical rainforest within the Barro Colorado Nature Monument; (2) forested islands (=I, six capture sites) situated in the Gatún Lake and also protected by the Barro Colorado Nature Monument, i.e., fragmented but otherwise undisturbed landscape; (3) fragmented and disturbed (i.e. contact to humans and domesticated animals) tropical lowland rainforest embedded in an agricultural matrix (=A, nine capture sites); and (4) fragmented and disturbed teak plantations (=P, seven capture sites) planted by humans and mainly consisting of *Tectona grandis*.

Field work took place during three field seasons (October 2013 to May 2014, October 2014 to May 2015 and September 2016 to April 2017, alternating the order of capture sites between seasons). At each capture site a trapping grid consisting of 100 stations was set up. Each station was separated by 20 m and consisted of three live traps [one Tomahawk trap (size: 15.2 × 15.2 × 48.3 cm, www.livetrap.com) and two Sherman traps (size: 10.2 × 11.4 × 38.1 cm, www.shermantraps.com)], one of which was placed above ground, if possible, i.e., on trees, lianas or similar, if available, to include arboreal species. Traps were opened at dusk and baited with a mixture of peanut butter, oatmeal, bird seeds, banana, and dog food to attract species with various dietary preferences and controlled at dawn of the next day. Then, captured animals were measured, individually marked to recognize recaptures, and fecal samples were taken from animals during sampling. Afterwards, the animals were released at the capturing location (further details see [24]). Fecal samples were stored in collection tubes containing RNAlater at − 20 °C until DNA extraction.

### DNA extraction, 16S rRNA gene amplification and sequencing

A detailed summary of sample processing is described elsewhere [17]. In brief, we extracted DNA from fecal samples from a total of 793 samples (including extraction blanks and PCR controls) using *NucleoSpin*^®^*Soil*-Extraction Kit from Macherey–Nagel (Germany). The final elution step was performed twice with 50 µl of elution buffer each time, resulting in a total volume of 100 µl. Following extraction, we amplified the 291 nucleotide-long V4 region of the 16S rRNA gene using the 515 F and 806 R primers [74, 75] applying a two-step polymerase chain reaction (PCR). The first step was an initial denaturation of 600 s at 95 °C followed by 30 cycles with 95 °C for 30 s, 60° for 30 s and 72 °C for 45 s, followed by a final elongation of 72 °C for 600 s. The second step consisted of ten cycles for the barcoding with the same conditions as described above. The samples were sequenced on six runs on an Illumina MiSeq at our Institute of Evolutionary Ecology and Conservation Genomics, Ulm University, Germany.

### Data processing

Reads from all six Illumina runs were analyzed in QIIME 2 (Version 2020.6, August 2020) [76] with a total of 14,489,625 sequences. DADA2 [77] was used to process the sequences and assemble these into amplicon sequence variants (ASVs) and SILVA (version 1.38) [78] was used as a taxonomic reference database. We removed a total of 194,432 sequences (roughly 0.83%) annotated as Archaea, mitochondria and chloroplast. Data were transferred into a *phyloseq* object [79] within the R environment (version 4.0.2) [80] for further analyses. ASVs identified in the blanks and controls (a total of 55,463 ASVs, equivalent to 0.38%) were removed from samples to avoid false results. We applied an additional filter to remove rare ASVs with fewer than 50 reads across the entire dataset and which occurred in only 2% of all the samples. Finally, we rarefied the data in order to control for uneven sequencing depth. Rarefaction was performed using the *rarefy_even_depth* function from the *phyloseq* package and a sequencing depth of 10,000 reads was chosen based on rarefaction curves (Additional file 1: Fig. S3). After performing all the bioinformatic quality filtering steps at rarefaction, 569 samples (*P. semispinosus*: *n* = 397 individuals; *D. marsupialis*: *n* = 104 individuals; *P. opossum*: *n* = 68) remained for subsequent analysis (Additional file 2: Table S1).

### Statistical analyses

Statistical analyses were separated into two parts based on the study questions. First, to determine if phylogeny and/or landscape affect the gut microbial beta diversity in the three species, we used the whole dataset, consisting of all three species and all four landscapes. Using weighted and unweighted UniFrac distances [81], we tested for differences in gut microbial composition and homogeneity according to landscape and host species using PERMANOVA (Permutational Analysis of Variance, with 9999 permutations) and PERMDISP2 (Permutational Analysis of Multivariate Dispersions) [82] using the *vegan* package [83]. These tests were followed by post-hoc pairwise comparisons. Effect sizes (Cohen’s *d*) [84] for pairwise comparisons were calculated by extracting the results from the PERMANOVA of the first two NMDS axes.

Second, to determine if the gut microbiome in each of the three host species is similarly impacted by landscape type, we subset our data for each species. Both alpha diversity (observed number of ASVs, Shannon Diversity and Faith’s phylogenetic diversity, PD) and beta diversity metrics (weighted and unweighted UniFrac distances) were calculated. For alpha diversity, we constructed generalized linear models (GLMs) with landscape, season, and sex as factors explaining the alpha diversity indices and afterwards used contrasts to analyze pairwise comparisons. For beta diversity, we applied PERMANOVAs and PERMDISPs as described above, followed by post-hoc pairwise comparisons calculated for each landscape.

Finally, to investigate which taxa were driving the differences in beta diversity, we performed differential abundance analyses using ANCOM (Analysis of Composition of Microbiomes) [39] which allows two-level factor comparisons [17]. We chose a *w*_*0*_ of 0.7 as originally described in [39]. Based on the results of the species-specific landscape effects on beta diversity (see Results), we compared the landscapes C + I + A versus P for *D. marsupials*, C versus A + P for *P. opossum* and C + I versus A + P for *P. semispinosus* for our two-level factor comparisons. All graphs were plotted in the R environment using the ggplot2 package [85].

## Supplementary Information

**Additional file 1: Fig. S1.** Location of the study area and 28 capture sites distributed across four landscapes differing in their anthropogenic impact in central Panama. Capture sites in the protected continuous tropical forests (landscape C) are marked in green; sites in the protected forested islands in the Panama Canal (landscape I) are marked in blue; sites in the nearby unprotected forested fragments embedded in an agricultural matrix (landscape A) are marked in yellow; and sites in teak plantations (landscape P) are marked in red. Map created with the R package ggmap (Kahle and Wickham, 2013) with the origin of the map material being Google Maps. **Fig. S2.** Distribution of the captured species across the four landscapes. Details on the landscapes C, I, A and P are provided in the methods and their locations are shown in Additional file 1: Fig. S1. **Fig. S3.** Rarefaction curves showing the number of detected ASVs in relation to 16S rRNA gene sequencing depth (i.e. total number of reads obtained per individual after quality filtering) for* Didelphis marsupialis* (turquoise), *Philander opossum* (orange) and *Proechimys semispinosus* (light-blue). The maximum diversity is reached at around 10,000 reads (vertical line). **Fig. S4.** Shared ASVs between *Didelphis marsupialis* (turquoise), *Philander opossum* (orange) and *Proechimys semispinosus* (light-blue).

**Additional file 2: Table S1.** Number of samples per species and landscape in the final dataset. Details on the landscapes C, I, A and P are provided in the methods and their locations are shown in Additional file 1: Fig. S1. **Table S2.** Effects of landscape type on the gut bacterial diversity of *D. marsupialis*. Results from generalized linear models indicating the effects of landscape type, field season and sex on alpha diversity using **a** Faith’s PD; **b** Number of ASVs and **c** Shannon diversity. Results from pairwise comparisons (Contrasts) of landscapes using **d** Faith’s PD; **e** Number of ASVs and **f** Shannon diversity. **g** Results from PERMANOVA for pairwise comparisons of landscapes on beta diversity (weighted and unweighted UniFrac). *SE* Standard error; *df* Degrees of freedom. **Table S3.** Effects of landscape type on the gut bacterial diversity of *P. opossum*. Results from generalized linear models indicating the effects of landscape type, field season and sex on alpha diversity using **a** Faith’s PD; **b** Number of ASVs and **c** Shannon diversity. Results from pairwise comparisons (Contrasts) of landscapes using **d** Faith’s PD; **e** Number of ASVs and **f** Shannon diversity. **g** Results from PERMANOVA for pairwise comparisons of landscapes on beta diversity (weighted and unweighted UniFrac). *SE* Standard error; *df* Degrees of freedom. **Table S4.** Effects of landscape type on the gut bacterial diversity of *P. semispinosus*. Results from generalized linear models indicating the effects of landscape type, field season and sex on alpha diversity using **a** Faith’s PD; **b** Number of ASVs and **c** Shannon diversity. Results from pairwise comparisons (Contrasts) of landscapes using **d** Faith’s PD; **e** Number of ASVs and **f** Shannon diversity. **g** Results from PERMANOVA for pairwise comparisons of landscapes on beta diversity (weighted and unweighted UniFrac). *SE* Standard error; *df* Degrees of freedom.

## Data Availability

The datasets generated and/or analyzed during the current study will be made available in upon acceptance.
