# Supplementary material for: Wildlife gut microbiomes of sympatric generalist species respond differently to anthropogenic landscape disturbances
Source: Anim Microbiome. 2023 Apr 6;5:22. doi: 10.1186/s42523-023-00237-9 (PMC10080760; doi:10.1186/s42523-023-00237-9)
Supplement: Supplementary file 1 — Additional file 1: Fig. S1. Location of the study area and 28 capture sites distributed across four landscapes differing in their anthropogenic impact in central Panama. Capture sites in the protected continuous tropical forests (landscape C) are marked in green; sites in the protected forested islands in the Panama Canal (landscape I) are marked in blue; sites in the nearby unprotected forested fragments embedded in an agricultural matrix (landscape A) are marked in yellow; and sites in teak plantations (landscape P) are marked in red. Map created with the R package ggmap (Kahle and Wickham, 2013) with the origin of the map material being Google Maps. Fig. S2. Distribution of the captured species across the four landscapes. Details on the landscapes C, I, A and P are provided in the methods and their locations are shown in Additional file 1: Fig. S1. Fig. S3. Rarefaction curves showing the number of detected ASVs in relation to 16S rRNA gene sequencing depth (i.e. total number of reads obtained per individual after quality filtering) for Didelphis marsupialis (turquoise), Philander opossum (orange) and Proechimys semispinosus (light-blue). The maximum diversity is reached at around 10,000 reads (vertical line). Fig. S4. Shared ASVs between Didelphis marsupialis (turquoise), Philander opossum (orange) and Proechimys semispinosus (light-blue). [file 42523_2023_237_MOESM1_ESM.pdf]

Supplementary Figures

Fig. S1

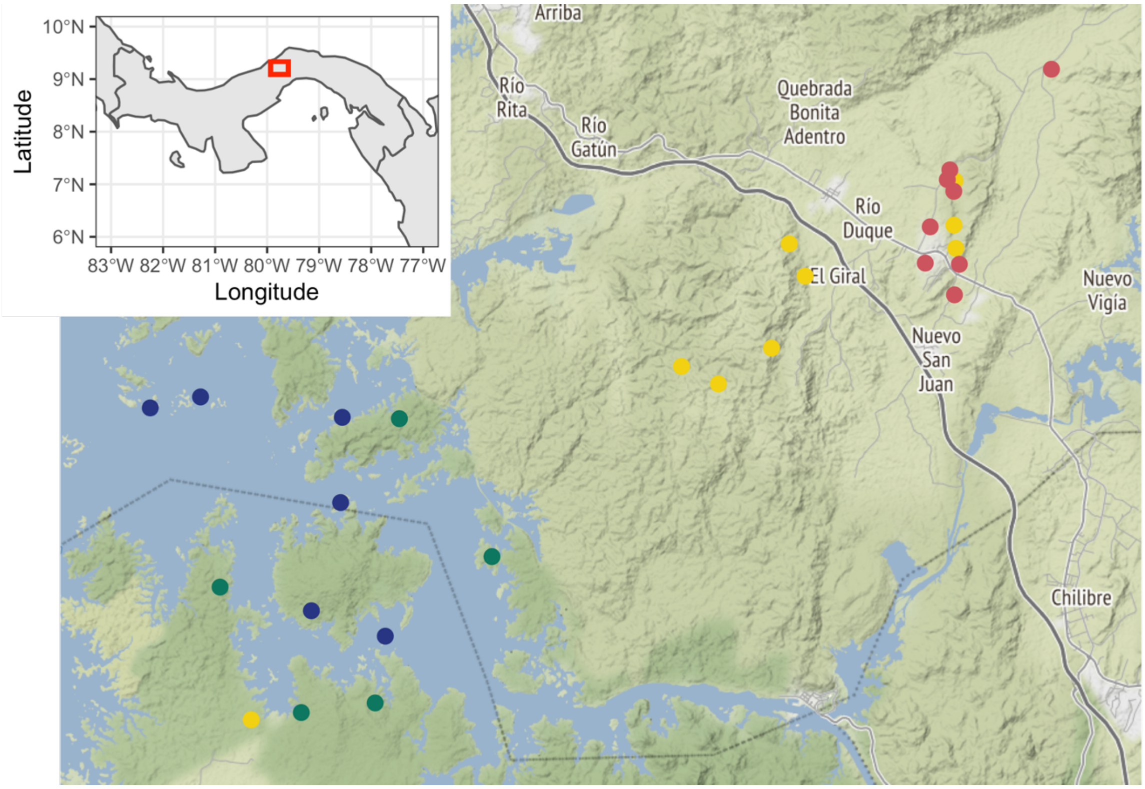

Supplementary Figure S1: Location of the study area and 28 capture sites distributed across four landscapes differing in their anthropogenic impact in central Panama. Capture sites in the protected continuous tropical forests (landscape C) are marked in green; sites in the protected forested islands in the Panama Canal (landscape I) are marked in blue; sites in the nearby unprotected forested fragments embedded in an agricultural matrix (landscape A) are marked in yellow; and sites in teak plantations (landscape P) are marked in red. Map created with the R package ggmap (Kahle and Wickham, 2013) with the origin of the map material being Google Maps.

Fig. S2

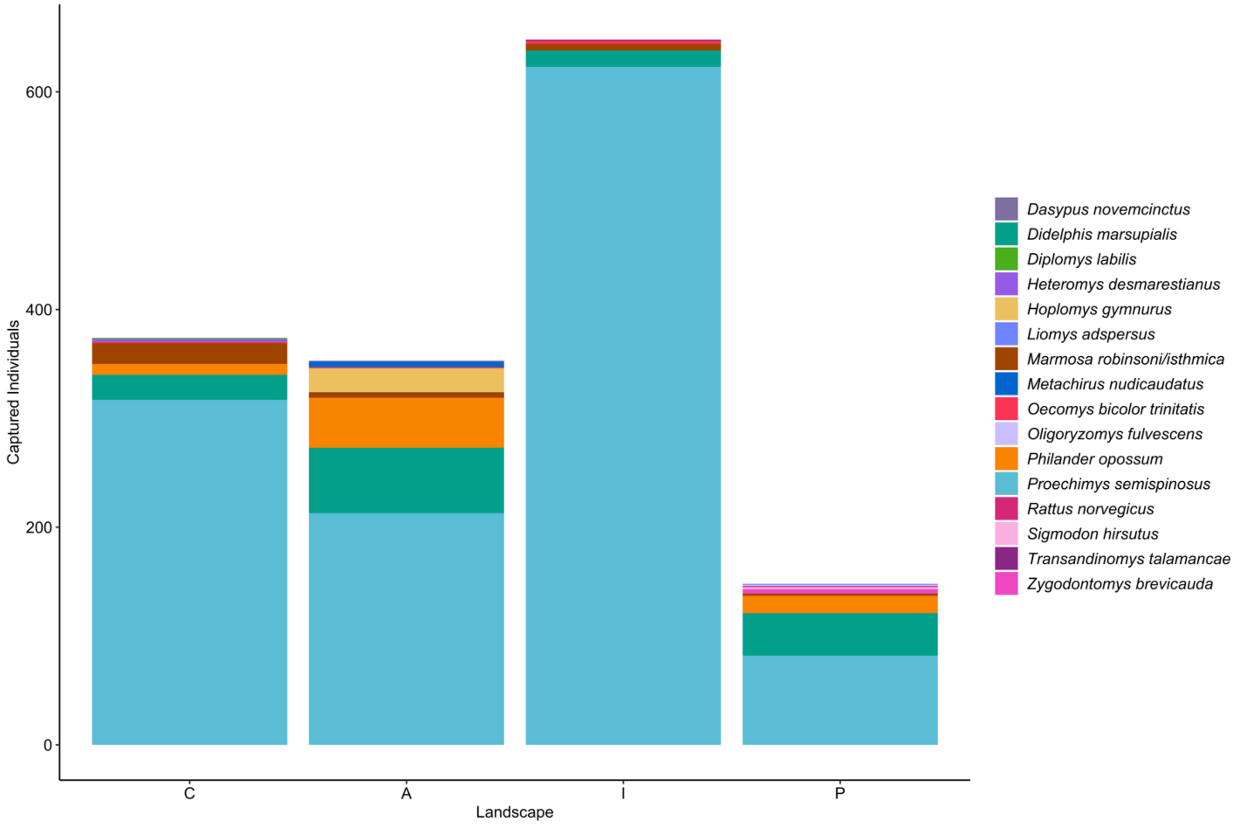

Supplementary Figure S2: Distribution of the captured species across the four landscapes. Details on the landscapes C, I, A and P are provided in the methods and their locations are shown in Fig. S1.

Fig. S3

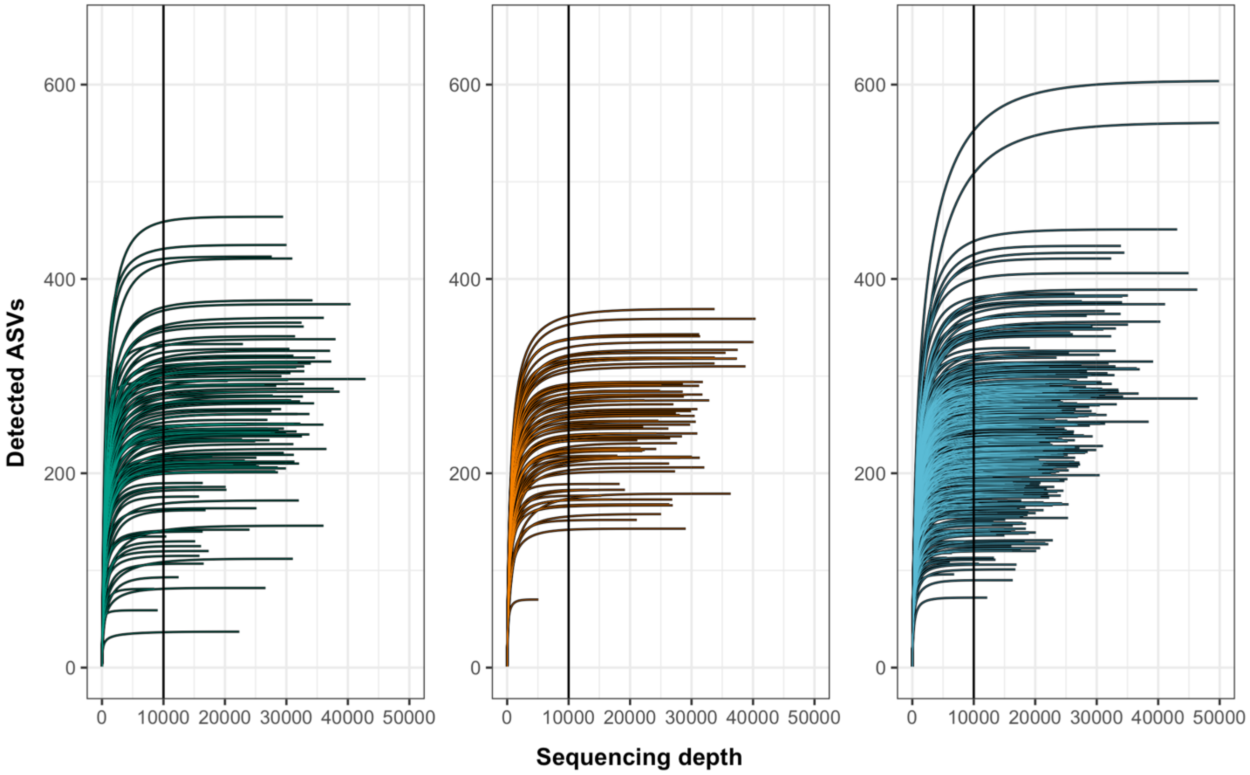

Supplementary Figure S3: Rarefaction curves showing the number of detected ASVs in relation to 16S rRNA gene sequencing depth (i.e. total number of reads obtained per individual after quality filtering) for *Didelphis marsupialis* (turquoise), *Philander opossum* (orange) and *Proechimys semispinosus* (light-blue). The maximum diversity is reached at around 10 000 reads (vertical line).

Fig. S4

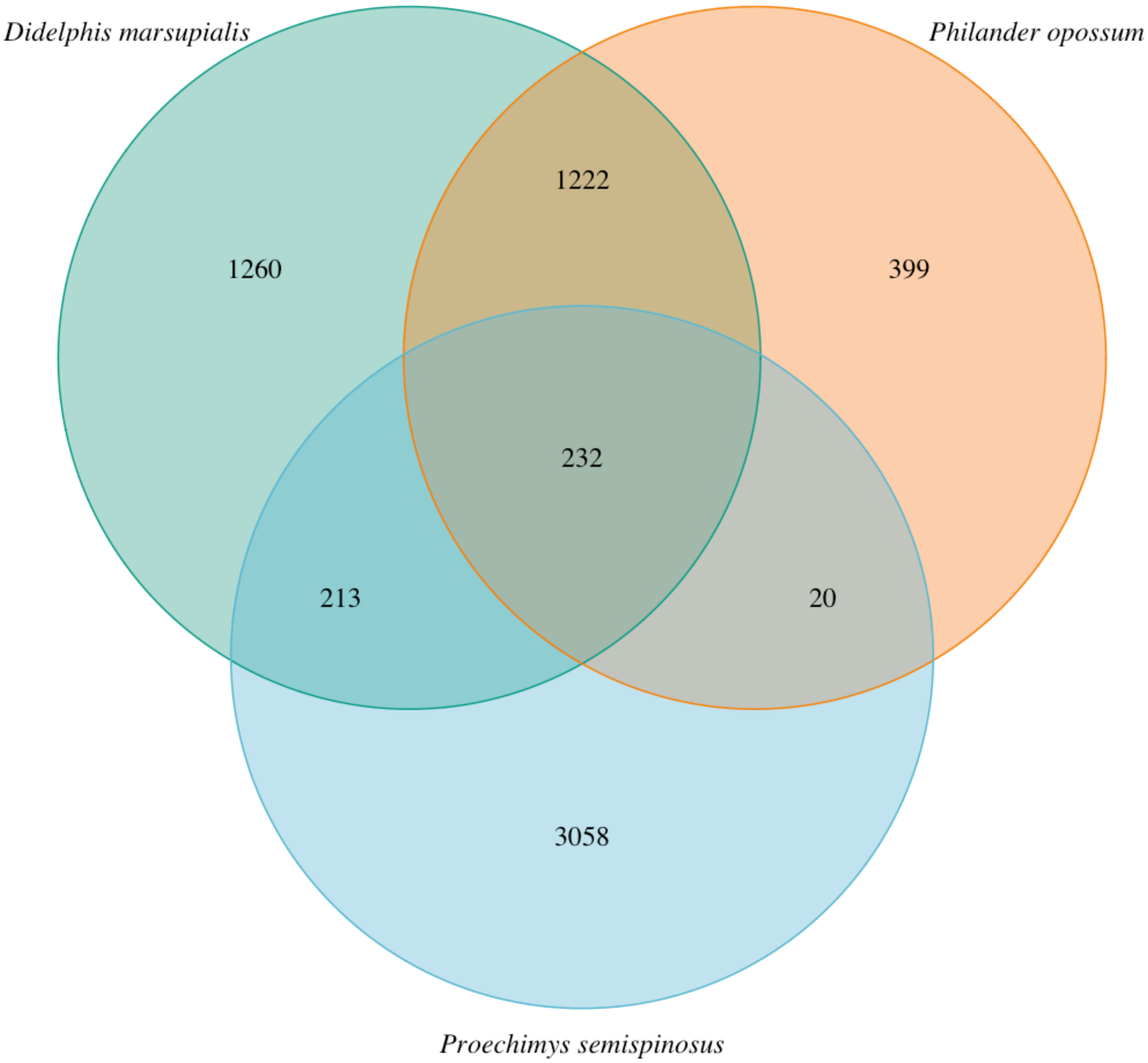

Supplementary Figure S4: Shared ASVs between *Didelphis marsupialis* (turquoise), *Philander opossum* (orange) and *Proechimys semispinosus* (light-blue).
