# Supplementary material for: Wildlife gut microbiomes of sympatric generalist species respond differently to anthropogenic landscape disturbances
Source: Anim Microbiome. 2023 Apr 6;5:22. doi: 10.1186/s42523-023-00237-9 (PMC10080760; doi:10.1186/s42523-023-00237-9)
Supplement: Supplementary file 2 — Additional file 2: Table S1. Number of samples per species and landscape in the final dataset. Details on the landscapes C, I, A and P are provided in the methods and their locations are shown in Additional file 1: Fig. S1. Table S2. Effects of landscape type on the gut bacterial diversity of D. marsupialis. Results from generalized linear models indicating the effects of landscape type, field season and sex on alpha diversity using a Faith’s PD; b Number of ASVs and c Shannon diversity. Results from pairwise comparisons (Contrasts) of landscapes using d Faith’s PD; e Number of ASVs and f Shannon diversity. g Results from PERMANOVA for pairwise comparisons of landscapes on beta diversity (weighted and unweighted UniFrac). SE Standard error; df Degrees of freedom. Table S3. Effects of landscape type on the gut bacterial diversity of P. opossum. Results from generalized linear models indicating the effects of landscape type, field season and sex on alpha diversity using a Faith’s PD; b Number of ASVs and c Shannon diversity. Results from pairwise comparisons (Contrasts) of landscapes using d Faith’s PD; e Number of ASVs and f Shannon diversity. g Results from PERMANOVA for pairwise comparisons of landscapes on beta diversity (weighted and unweighted UniFrac). SE Standard error; df Degrees of freedom. Table S4. Effects of landscape type on the gut bacterial diversity of P. semispinosus. Results from generalized linear models indicating the effects of landscape type, field season and sex on alpha diversity using a Faith’s PD; b Number of ASVs and c Shannon diversity. Results from pairwise comparisons (Contrasts) of landscapes using d Faith’s PD; e Number of ASVs and f Shannon diversity. g Results from PERMANOVA for pairwise comparisons of landscapes on beta diversity (weighted and unweighted UniFrac). SE Standard error; df Degrees of freedom. [file 42523_2023_237_MOESM2_ESM.pdf]

Supplementary Tables

Supplementary Table S1: Number of samples per species and landscape in the final dataset. Details on the landscapes C, I, A and P are provided in the methods and their locations are shown in Fig. S1.

Species	C	I	A	P
D. marsupialis	21	10	42	31
P. opossum	10	0	43	15
P. semispinosus	94	129	136	38

Supplementary Table S2: Effects of landscape type on the gut bacterial diversity of *D. marsupialis*. Results from generalized linear models indicating the effects of landscape type, field season and sex on alpha diversity using a) Faith's PD); b) Number of ASVs and c) Shannon diversity. Results from pairwise comparisons (Contrasts) of landscapes using d) Faith's PD; e) Number of ASVs and f) Shannon diversity. g) Results from PERMANOVA for pairwise comparisons of landscapes on beta diversity (weighted and unweighted UniFrac). SE = standard error; df = degrees of freedom.

a) Faith's PD

Coefficients:

	Estimate	SE	t value	Pr(>|t|)	
(Intercept)	2.99	0.07	41.51	< 2e-16	***
A	0.04	0.06	0.69	0.49	
I	0.17	0.08	2.09	0.04	*
P	-0.25	0.07	-3.5	<0.001	***
2014/15	0.12	0.07	1.82	0.07	.
2016/17	0.02	0.07	0.23	0.82	
Male	0.01	0.05	0.15	0.88	

b) Number of ASVs

Coefficients:

	Estimate	SE	t value	Pr(>|t|)	
(Intercept)	5.47	0.09	63.87	< 2e-16	***
A	0.08	0.07	1.05	0.3	
I	0.25	0.1	2.55	0.01	*
P	-0.26	0.09	-2.87	<0.01	**
2014/15	0.06	0.08	0.81	0.42	
2016/17	-0.01	0.09	-0.11	0.92	
Male	0	0.06	-0.09	0.93	

c) Shannon diversity

Coefficients:

	Estimate	SE	t value	Pr(>|t|)	
(Intercept)	1.45	0.04	34.26	<2e-16	***
A	0.05	0.04	1.3	0.2	
I	0.12	0.05	2.35	0.02	*
P	-0.08	0.04	-1.95	0.05	.
2014/15	0	0.04	-0.09	0.93	
2016/17	-0.02	0.04	-0.36	0.72	
Male	-0.01	0.03	-0.48	0.63	

d) Contrasts Faith's PD

Contrast	Estimate	SE	df	z.ratio	P
C – A	-0.04	0.06	Inf	-0.69	0.49
C – I	-0.17	0.08	Inf	-2.09	0.06
C – P	0.25	0.07	Inf	3.5	<0.001
A – I	-0.13	0.08	Inf	-1.69	0.11
A – P	0.3	0.06	Inf	4.82	<0.001
I – P	0.42	0.09	Inf	4.84	<0.001

e) Contrasts number of ASVs

Contrast	Estimate	SE	df	z.ratio	P
C – A	-0.08	0.07	Inf	-1.05	0.3
C – I	-0.25	0.1	Inf	-2.55	0.02
C – P	0.26	0.09	Inf	2.87	<0.01
A – I	-0.17	0.09	Inf	-1.89	0.07
A – P	0.34	0.08	Inf	4.42	<0.001
I – P	0.51	0.1	Inf	4.84	<0.001

f) Contrasts Shannon diversity

Contrast	Estimate	SE	df	z.ratio	P
C – A	-0.05	0.04	Inf	-1.3	0.19
C – I	-0.12	0.05	Inf	-2.35	0.04
C – P	0.08	0.04	Inf	1.95	0.08
A – I	-0.07	0.05	Inf	-1.48	0.17
A – P	0.13	0.04	Inf	3.73	<0.001
I – P	0.2	0.05	Inf	3.86	<0.001

g) Beta diversity

Comparison	weighted UniFrac	unweighted UniFrac
C – F	0.553	0.135
C – I	0.526	0.135
C – P	0.514	0.03
F – I	0.526	0.208
F – P	0.072	0.013
I – P	0.269	0.03

Supplementary Table S3 Effects of landscape type on the gut bacterial diversity of *P. opossum*. Results from generalized linear models indicating the effects of landscape type, field season and sex on alpha diversity using a) Faith's PD); b) Number of ASVs and c) Shannon diversity. Results from pairwise comparisons (Contrasts) of landscapes using d) Faith's PD; e) Number of ASVs and f) Shannon diversity. g) Results from PERMANOVA for pairwise comparisons of landscapes on beta diversity (weighted and unweighted UniFrac). SE = standard error; df = degrees of freedom.

a) Faith's PD

Coefficients:

	Estimate	SE	t value	Pr(>|t|)	
(Intercept)	2.86	0.15	18.89	<2e-16	***
A	0.07	0.07	1.05	0.3	
P	0.02	0.08	0.33	0.74	
2014/15	0.02	0.07	0.28	0.78	
2016/17	-0.03	0.07	-0.41	0.69	
Female	0.04	0.14	0.3	0.76	
Male	0.07	0.13	0.53	0.6	

b) Number of ASVs

Coefficients:

	Estimate	SE	t value	Pr(>|t|)	
(Intercept)	5.34	0.21	25.48	<2e-16	***
A	0.1	0.09	1.21	0.23	
P	0.1	0.1	1.06	0.3	
2014/15	-0.06	0.08	-0.79	0.43	
2016/17	-0.09	0.08	-1.08	0.29	
Female	0.14	0.19	0.72	0.48	
Male	0.16	0.19	0.83	0.41	

c) Shannon diversity

Coefficients:

	Estimate	SE	t value	Pr(>|t|)	
(Intercept)	1.49	0.09	17.38	<2e-16	***
A	0.08	0.04	1.97	0.05	.
P	0.1	0.04	2.34	0.02	*
2014/15	-0.07	0.04	-1.72	0.09	.
2016/17	-0.05	0.04	-1.22	0.23	
Female	-0.02	0.08	-0.3	0.76	
Male	-0.02	0.08	-0.3	0.76	

d) Contrasts Faith's PD

Contrast	Estimate	SE	df	z.ratio	P
C – A	-0.07	0.07	Inf	-1.05	0.65
C – P	-0.02	0.08	Inf	-0.33	0.74
A – P	0.04	0.06	Inf	0.79	0.65

e) Contrasts number of ASVs

Contrast	Estimate	SE	df	z.ratio	P
C – A	-0.1	0.09	Inf	-1.21	0.44
C – P	-0.1	0.1	Inf	-1.06	0.44
A – P	0	0.07	Inf	0.03	0.98

f) Contrasts Shannon diversity

Contrast	Estimate	SE	df	z.ratio	P
C – A	-0.08	0.04	Inf	-1.97	0.07
C – P	-0.1	0.04	Inf	-2.34	0.06
A – P	-0.03	0.03	Inf	-0.82	0.41

g) Beta diversity

Comparison	weighted UniFrac	unweighted UniFrac
C – F	0.26	0.014
C – P	0.26	0.013
F – P	0.45	0.037

Supplementary Table S4: Effects of landscape type on the gut bacterial diversity of *P. semispinosus*. Results from generalized linear models indicating the effects of landscape type, field season and sex on alpha diversity using a) Faith's PD); b) Number of ASVs and c) Shannon diversity. Results from pairwise comparisons (Contrasts) of landscapes using d) Faith's PD; e) Number of ASVs and f) Shannon diversity. g) Results from PERMANOVA for pairwise comparisons of landscapes on beta diversity (weighted and unweighted UniFrac). SE = standard error; df = degrees of freedom.

a) Faith's PD

Coefficients:

	Estimate	SE	t value	Pr(>|t|)	
(Intercept)	2.89	0.07	39.29	< 2e-16	***
A	-0.12	0.02	-5.85	<0.001	***
I	0	0.02	-0.14	0.89	
P	-0.12	0.03	-3.82	<0.001	***
2014/15	0.07	0.02	3.14	<0.01	**
2016/17	-0.01	0.02	-0.63	0.53	
Female	-0.06	0.07	-0.89	0.37	
Male	-0.06	0.07	-0.84	0.4	

b) Number of ASVs

Coefficients:

	Estimate	SE	t value	Pr(>|t|)	
(Intercept)	5.7	0.11	53.88	< 2e-16	***
A	-0.23	0.03	-6.94	<0.001	***
I	0.01	0.03	0.34	0.74	
P	-0.23	0.05	-4.39	<0.001	***
2014/15	0.12	0.04	3.3	<0.01	**
2016/17	0	0.04	-0.08	0.94	
Female	-0.2	0.1	-2.01	<0.05	*
Male	-0.17	0.1	-1.76	0.08	.

c) Shannon diversity

Coefficients:

	Estimate	SE	t value	Pr(>|t|)	
(Intercept)	1.57	0.04	35.99	< 2e-16	***
A	-0.08	0.01	-6.41	<0.001	***
I	-0.01	0.01	-0.61	0.55	
P	-0.06	0.02	-3.29	<0.01	**
2014/15	0.01	0.01	0.43	0.67	
2016/17	0.01	0.01	0.62	0.54	
Female	-0.07	0.04	-1.72	0.09	.
Male	-0.07	0.04	-1.59	0.11	

d) Contrasts Faith's PD

Contrast	Estimate	SE	df	z.ratio	P
C – A	0.12	0.02	Inf	5.85	<0.001
C – I	0	0.02	Inf	0.14	0.98
C – P	0.12	0.03	Inf	3.82	<0.001
A – I	-0.12	0.02	Inf	-5.98	<0.001
A – P	0	0.03	Inf	0.02	0.98
I – P	0.12	0.03	Inf	3.86	<0.001

e) Contrasts number of ASVs

Contrast	Estimate	SE	df	z.ratio	P
C – A	0.23	0.03	Inf	6.94	<0.001
C – I	-0.01	0.03	Inf	-0.34	0.89
C – P	0.23	0.05	Inf	4.39	<0.001
A – I	-0.24	0.03	Inf	-7.58	<0.001
A – P	0	0.05	Inf	0.04	0.97
I – P	0.24	0.05	Inf	4.73	<0.001

f) Contrasts Shannon diversity

Contrast	Estimate	SE	df	z.ratio	P
C – A	0.07616	0.0119	Inf	6.405	<0.001
C – I	0.01	0.01	Inf	0.61	0.54
C – P	0.06	0.02	Inf	3.29	<0.01
A – I	-0.07	0.01	Inf	-6.14	<0.001
A – P	-0.02	0.02	Inf	-1.14	0.31
I – P	0.05	0.02	Inf	2.99	<0.01

g) Beta diversity

Comparison	weighted UniFrac	unweighted UniFrac
C – F	0.001	0.001
C – I	0.001	0.001
C – P	0.001	0.001
F – I	0.001	0.001
F – P	0.001	0.001
I – P	0.001	0.001
